# Confronting Passive and Active Sensors with Non-Gaussian Statistics

**DOI:** 10.3390/s140813759

**Published:** 2014-07-30

**Authors:** Pablo. Rodríguez-Gonzálvez, Jesús. Garcia-Gago, Javier. Gomez-Lahoz, Diego. González-Aguilera

**Affiliations:** Department of Cartographic and Land Engineering, University of Salamanca, Polytechnic School of Avila. Hornos Caleros, 50, 05003, Avila, Spain

**Keywords:** passive sensor, active sensor, digital camera, laser scanner, non-Gaussian statistic, non-parametric statistic, measurement

## Abstract

This paper has two motivations: firstly, to compare the Digital Surface Models (DSM) derived by passive (digital camera) and by active (terrestrial laser scanner) remote sensing systems when applied to specific architectural objects, and secondly, to test how well the Gaussian classic statistics, with its Least Squares principle, adapts to data sets where asymmetrical gross errors may appear and whether this approach should be changed for a non-parametric one. The field of geomatic technology automation is immersed in a high demanding competition in which any innovation by one of the contenders immediately challenges the opponents to propose a better improvement. Nowadays, we seem to be witnessing an improvement of terrestrial photogrammetry and its integration with computer vision to overcome the performance limitations of laser scanning methods. Through this contribution some of the issues of this “technological race” are examined from the point of view of photogrammetry. A new software is introduced and an experimental test is designed, performed and assessed to try to cast some light on this thrilling match. For the case considered in this study, the results show good agreement between both sensors, despite considerable asymmetry. This asymmetry suggests that the standard Normal parameters are not adequate to assess this type of data, especially when accuracy is of importance. In this case, standard deviation fails to provide a good estimation of the results, whereas the results obtained for the Median Absolute Deviation and for the Biweight Midvariance are more appropriate measures.

## 1. Introduction

### 1.1. Motivation

There is little doubt that use of photogrammetry has undergone a resurgence in its duel with the powerful laser scanning systems, both terrestrial (TLS-Terrestrial Laser Scanner) and aerial (ALS-Airborne Laser Scanner), and both static and dynamic (MMS-Mobile Mapping System) [1]. During the second half of the last decade, the latter seemed to pose an insurmountable challenge for the former technology which looked almost decadent, but quite surprisingly, we are witnessing a return of photogrammetry in the geomatic market, where nothing is yet decided.

The secret of the success of the recent photogrammetry proposals is based on two issues: firstly, its bid to continue trusting/relying on the strength of its information source, the image; secondly, its “marriage” with its “relative”, computer vision [2]. This hybridization has enabled the discipline to overcome two important hurdles. The first one has been surpassed by the automation of the orientation and reconstruction processes. This led to highly dense three dimensional models that in many cases surpass the resolution of laser scanning ones. The second obstacle was related to the classical rigid geometric configuration in the shooting network, namely, the so called, normal case. Nowadays, this configuration has been substituted by a highly flexible one. In addition, any type of sensor, calibrated and non-calibrated [3], or even smartphone or tablet devices [4] can be used. Besides these two recent performance developments, there is a third issue that has characterized photogrammetry over the years as a metrology discipline: its compromise with respect to accuracy in order to increase reliability [3]. This traditional line has yielded results that, in many applications, can stand as a low cost competitor to the laser systems.

On the other hand, but no less important, we are aided by a proliferation of multipurpose, low cost or even free, open source tools that deal with multiple image processing to render 3D geometric and radiometric models. This challenge requires solving two previous important geometric issues: how to compute the camera parameters (interior orientation, principal distance or focal length, coordinates of the principal point, and radial and tangential distortion parameters) and how to compute the shooting parameters (exterior orientation, world coordinates of the point of view and angular parameters—rotation matrix—of the camera axis when shooting) [4]. For years, photogrammetry addressed these problems by applying rigorous laboratory calibration protocols and later with robust and accurate control points networks, but the implementation and fusion of algorithms and techniques largely developed on the computer vision community has led to the automatic computation of these items while computing the 3D model itself.

More specifically, great advances have occured in the use of robust strategies for image matching based on features (FBM-Feature Based Matching) and on areas (ABM-Area Based Matching) that enable the task of linking the images to one another or to some external element and thus, making available the transition from the 2D information to the 3D information, an issue that was traditionally performed through stereoscopic skills.

As is well known this is the basis of photogrammetry since it can enable the computation of the interior and relative exterior orientations and, at the same time, the computation of a sparse model of the object on a local and arbitrary frame [5]. A certain knowledge of the frame (through control points or through an explicit datum definition), enables the application of the colinearity equations [6] that leads to an absolute model location through: (a) the reverse intersection problem that renders the absolute exterior orientation of the images and (b) the forward intersection problem that yields the absolute coordinates of any object point from corresponding image sets (at least two) of points.

In this regard, very important and powerful innovations in the field of matching have been achieved in the last years. Firstly there is the powerful but dependent on a favourable geometry (photogrammetric normal case) semi-global matching (SGM) strategy [7] which permits rendering of high density 3D point clouds with resolutions up to the Ground Sampling Distance (GSD), that is, one 3D object point for each pixel of the image. Apero-Micmac [8] is a good example of Open Source software beside commercial photogrammetric tools (eATE, NGATE,Dense Matcher, ISAE, Match-T, Xpro, Tridicon, *etc.*). In any case, other important achievements exist in the case of unfavourable geometry, such as those patch-based methods supported by surfels [9–11]. In this sense, Patch-based Multi-view Stereo (PMVS) assumes a hybrid approach, using undistorted images, orientation parameters of the images, a sparse set of points and the projection matrixes to determine a dense set of rectangular patches. Nevertheless, the major drawback of these commercial and web-based tools (Bundler, Photosynth, Photofly, PMVS, *etc.*) is that they are only based on computer vision algorithms and thus they do not provide accurate results. Last but not least, an additional drawback for these web-based methods is their computation cost, especially if large format cameras and high resolution images are involved. All these considerations suggest the need for further research towards the implementation of parallel processing techniques and the use of the graphical memory in order to minimize the processing effort.

One of the main goals of this paper is to present the Photogrammetry Workbench (PW) software as an attempt to reinforce the relation between photogrammetry and computer vision in the context of the Heritage Architecture field according to the considerations stated above, but an even more important motivation is to implement and assess some statistical tools to improve the processing of data that does not behave in a Gaussian fashion, which is the usual case when dealing with automated point clouds, regardless of whether they are generated by photogrammetry or laser scanning.

About 15 years ago, Kraus coined the motto “quantity *vs.* quality”. He did so to try to illustrate the change of paradigm that digital photogrammetry was leading. Traditional photogrammetry was very much based on quality, that is, on human skill, and therefore, on Gaussian statistics. As is well known [3], the redundant display that underlies aerial triangulation or the bundle adjustment is solved through the Least Squares Criteria that assumes that the image observations follow a normal distribution, namely, they can be represented by means of a standard deviation. This was representative of the human ability to perform good but few observations (redundancy not higher than 50). Contrary to this manual paradigm is the automatic one that relies heavily on the computer's capacity to perform poor but abundant observations, that is, based on quantity. This means that besides a high number of correct redundant observations (many more than 100) the process is plagued by a number of gross errors (outliers) and consequently, the mean—standard deviation—least squares assumptions must be revised.

### 1.2. Objectives

The main objectives of this article were to test the comparison of photogrammetric and laser scanning point clouds and to test the performance of Gaussian and non-Gaussian statistics when comparing data sets. For this purpose an experiment was designed in which two Romanesque portals were captured by means of two laser scanning systems and photogrammteric systems (digital cameras). The photogrammetric processing of the images was done by means of two software packages: one developed in-house by the authors and a well-known commercial software (see Section 4 for a more detailed description of the experiment).

## 2. From a Set of 2D Images to a Dense 3D Point Cloud

The software here presented, PW, is a multiplatform software which integrates photogrammetric robust algorithms with automatic and flexible approaches coming from computer vision operating behind a user-friendly interface that works with terrestrial or aerial images and with vertical or oblique geometries. This software works on two parts that correspond to the classical main steps in photogrammetry: orientation and modelling. The input of the first part (orientation) is a dense set of matching points extracted from a set of images by means of a robust operator and the output is the exterior orientation parameters of these set of images. The input of the second part (modelling) are these set of orientation parameters and an optimized version of the same set of matching points used for the first part. The final output is a dense point cloud that can withstand the comparison with 3D laser scan point clouds.

### 2.1. Extraction of Matching Points from the Images

The task of finding matching features that appear in corresponding images, and the degree of variation between the patches of images to be compared are very important issues. On the most simple side of the wide range of approaches that have been implemented to deal with this question, we have the most classical matching method in photogrammetry: ABM [12]. This method works efficiently because it demands *a priori* that images have been taken under the classical geometric paradigm of aerial photogrammetry: the so called, normal case. As is well known, this implies, firstly, that images must be almost parallel to the object and thus, with no perspective effects between them. Secondly, the variation implies, secondly, that the variation of distance to the object must be kept under small thresholds (terrain surface, the object of aerial photogrammetry, is usually smooth). So, when dealing with images that exhibit important perspective variations or important scale variations, this method does not render acceptable results.

Thus, a variety of methods have been developed in recent years to handle these variations between images. It has been usually assumed that these variations can be modelled by a translation, a change in scale and a rotation. Some of these methods are Smallest Univalue Segment Assimilating Nucleus (SUSAN) [13], Scale Invariant Feature Transform (SIFT) [14], Maximally Stable Extremal Region (MSER) [15], or Speeded Up Robust Features (SURF) [16]. None of these algorithms are robust enough to cope with high perspective variations between images.

Affine Scale Invariant Transform (ASIFT) [17] is a method that is affine invariant on a local region patch and has proved to be robust enough to deal with images that present rather large perspective effects as a whole. In ASIFT, two rotation parameters are added to the plane rotation that is included in the methods mentioned above. This provides the two degrees of freedom that render the two angles of a line (camera axis) with respect to a plane (main object plane). Consequently, the algorithm enables one to work with perspective images, which are frequent in terrestrial photogrammetry.

### 2.2. Orientation Procedure

The matching points derived from the ASIFT operator are the input for the orientation procedure which is performed in two steps. In the first one, a pairwise orientation is executed by relating the images to each other by means of the fundamental matrix model. In the second one, this initial and partial approximation to the solution is used to perform a global bundle adjustment by means of the colinearity equations which include the determination of the camera parameters (self-calibration).

#### 2.2.1. Relative Orientation

The five degrees of freedom that underlie the non-linear equations (coplanarity equation [6] that relate whatever two images that share corresponding features can be expressed by a eight parameter linear model, based on the so called fundamental matrix [5] computed by the Longuet-Higgins algorithm [18] according to the equation:

(1) $${{\text{x}}^{\prime }\text{xf}}_{11}+{{\text{x}}^{\prime }\text{yf}}_{12}+{{\text{x}}^{\prime }\text{f}}_{13}+{{\text{y}}^{\prime }\text{xf}}_{21}+{{\text{y}}^{\prime }\text{yf}}_{22}+{{\text{y}}^{\prime }\text{f}}_{23}+{\text{xf}}_{31}+{\text{yf}}_{32}+{\text{f}}_{33}=0\;$$

where *f_ij_* are the terms of the 3 × 3 fundamental matrix and (*x*, *y*) and (*x′*, *y′*) are the image coordinates of every pair of matching points. Once these terms are obtained, the five geometric parameters that express the relative position (up to scale) and attitude of one image respect the other can be derived [19] provided an approximate description of the camera is known. In this way we obtain an initial estimation of a pairwise orientation of the images given an arbitrary datum.

#### 2.2.2. Bundle Adjustment

The knowledge derived from the previous section provides approximations good enough to solve the so called bundle adjustment [6] which permits to stablish a relationship between images and object by means of colinearity Equation (2) in a non-linear fashion, that is, an iterative approach of progressively better solutions until convergence is achieved:

(2) $$\begin{array}{ll} (x-x_{p})+\Delta x & =-f\frac{r_{11}(X-S_{X})+r_{21}(Y-S_{Y})+r_{31}(Z-S_{Z})}{r_{13}(X-S_{X})+r_{23}(Y-S_{Y})+r_{33}(Z-S_{Z})}\\ (y-y_{p})+\Delta y & =-f\frac{r_{12}(X-S_{X})+r_{22}(Y-S_{Y})+r_{32}(Z-S_{Z})}{r_{13}(X-S_{X})+r_{23}(Y-S_{Y})+r_{33}(Z-S_{Z})} \end{array}$$

where *x* and *y* are the image coordinates; *X*, *Y*, *Z* are the object coordinates; (*r_ij_*, *S_X_*, *S_Y_*, *S_Z_*) are the parameters of the exterior orientation of each image; (*x_p_*, *y_p_*, *f*) are the geometrical parameters of the interior orientation of the camera, that is, the image principal point coordinates and the focal length; (*ΔX*, *ΔY)* are the corrections to be applied to the image coordinates of an observed point because of the radial and tangential distortion of the lens according to Equation (3):

(3) $$\begin{array}{ll} \Delta x & =-x_{p}-\frac{x^{\prime }}{f}\Delta f+x^{\prime }(r^{2}k_{1}+r^{4}k_{2}+r^{6}k_{3})+(2x^{\prime 2}+r^{2})p_{1}+2p_{2}x^{\prime }y^{\prime }+b_{1}x^{\prime }+b_{2}y^{\prime }\\ \Delta y & =-y_{p}-\frac{y^{\prime }}{f}\Delta f+y^{\prime }(r^{2}k_{1}+r^{4}k_{2}+r^{6}k_{3})+2p_{1}x^{\prime }y^{\prime }+(2y^{\prime 2}+r^{2})p_{2} \end{array}$$

This is the Fraser model [20] which takes into account additional parameters to the Gaussian distortion model [21]: besides the principal distance (*f)*, and principal point coordinates (*x_p_*, *y_p_*), the parameters (*k_1_*, *k_2_*, *k_3_*) are used to render the radial distortion whereas (*p_1_*, *p_2_*) address the tangential distortion. Furthermore, it considers terms for affinity (*b_1_*) and non-orthogonality (*b_2_*).

Two important considerations must be taken into account: (a) the bundle adjustment can be carried on with or without the knowledge of the camera. In the first case the interior and the lens distortion parameters are entered as their known value. In the second case, they must be considered as unknowns (self-calibration) and solved with the whole set of unknowns; (b) this is the moment in which an absolute datum can be defined. This can be done by means of the object coordinates of ground control points expressed in whatever geodetic system or by means of geometric restrictions that define the seven parameters of the coordinate frame.

### 2.3. Modelling Procedure

Once the orientations step is completed, the whole set of images can be processed based on the epipolarization constraints [22], that is, for a given pixel on a given image the search space of the correspondent pixel in some other image can be computed as an (epipolar) line, thus minimizing the search cost. Relying on this principle a Digital Surface Model (DSM) can be computed by means of forward ray intersection [6]. To solve this process, a SGM (Semi-Global Matching) technique [7] has been developed. The final 3D coordinates are calculated using the Equation (4):

(4) $$X_{k}=C(D(R_{i}(x_{ik}-S_{i})))$$

where the *k*-th unknown ground point (***X_k_***) is related to its projection (***x_ik_*)** on an i-th image by means of the extrinsic parameters of this image parameterized by the rotation matrix (**R_i_**) and the known projection center of the image (***S_i_*)**. The intrinsic parameters are the camera matrix (***C***) and the lens distortion function (*D*).

## 3. Normal *vs.* Non-Normal Statistics

The automation of the matching task is a process where a high number of false correspondences occur. These errors must be filtered in successive steps to determine the orientation, but even though a good orientation solution may be achieved, that does not imply that the subsequent Digital Surface Model (DSM) is free from blunders since there may be correspondences that do not belong to the object but to alien elements such as vegetation, urban artefacts, traffic, people, birds, *etc.*

On the other hand, when comparing the DSM obtained by means of photogrammetry to the DSM obtained by means of laser scanning it is important to have in mind that both systems have their own sources of error and their own sensitivity to the impact of outliers. In addition, if both systems are to be compared, it is important to notice that the points on both DSMs are not the same and thus, a strategy to find the best correspondences must be applied. An additional source of gross error is that the positions of both sensors are not identical and thus, not all the object surfaces are equally captured in the data sets.

Due to the reasons mentioned above, it should be expected that the percentage of gross errors associated to the data sets increases and thus, the methods based on normal Gaussian statistic do not perform well. To do so, the steps to be applied are the following: firstly, to check the normality assumption based on statistical graphics (QQ-plots) and numerical methods (skewness and kurtosis indices). Secondly, to test the accuracy measures of discrepancies dataset for the normal distribution based on mean error (*μ*), standard deviation (*σ*) and their corresponding confidence intervals (*CI*). And thirdly, to apply a robust model based on non-parametric estimation using sample quantiles as reference and adding the median (*m*) and the biweight midvariance (*BWMV*) as robust measures of the mean and standard deviation, respectively.

Therefore, we propose a break with the “deeply rooted custom” of the zero and normal distribution of errors being considered as the appropriate standard measure of accuracy in the photogrammetric and laser scanning studies (e.g., as adopted by the National Standard for Spatial Data Accuracy—NSSDA), incorporating and establishing a comparison between parametric and nonparametric statistical methods.

Although the sensitivity of normality tests to non-normal data could seem an efficient alternative, it should be remarked that these tests do not work properly with large datasets since the central limit theorem comes into play [23], so that normality tests were only applied in those cases with a reduced number of observations. To large datasets, a better diagnostic for checking a deviation from the normal distribution is the visual plot quantile-quantile (QQ-plot). In this case, the quantiles of the empirical distribution function are plotted against the theoretical quantiles of the normal distribution. If the distribution follows a Gaussian function, the QQ-plot should be a diagonal straight line. The skewness parameter (Equation (5)) provides an indication of departure from symmetry in a distribution (asymmetry around the mean value), whereas kurtosis parameter (Equation (6)) is a measure of whether the data are peaked or flat relative to a normal distribution. If the distribution is perfectly normal, skewness and kurtosis values of zero are obtained:

(5) $$\text{Skewness}=\frac{\overset{n}{\underset{i=1}{\text{∑}}}{\left(x_{i}-\mu \right)}^{3}}{(n-1)\sigma^{3}}$$

(6) $$\text{Kurtosis}=\frac{\overset{\text{n}}{\underset{\text{i}=1}{\text{∑}}}{\left(x_{i}-\mu \right)}^{4}}{(n-1)\sigma^{4}}-3$$

where *μ* is the mean, *n* is the number of data points and *σ* is the standard deviation.

When the datasets follow a normal distribution the classical accuracy measures such as mean error (*μ*) and standard deviation (*σ*) are considered. Likewise, confidence intervals (*CI*) are provided for parameters based on the theory of errors and considering the interval (*x* ± 1.96*σ*), where *x* is the parameter and *σ* its standard deviation for a confidence level of 95%. In those cases where some minimum outliers could remain, the parametric approaches establish the 3*σ* rule to remove outliers which can corrupt the true statistical distribution of errors. If after applying the 3*σ* rule error data sets still follow a non-normal distribution, robust and non-parametric methods for the derivation of accuracy measures should therefore be applied. For a small Gaussian sample size, the 3*σ* rule could be replaced by Chauvenet's criterion [24] that rejects those errors which have a probability of occurrence that is less than the probability of occurrence corresponding to a proportion of 1 − 1/(4*n*) of the sample, being *n* the sample size [25]. For those cases for which the distribution of the data is not known, other approaches for deriving accuracy measures need to be applied. Interquartile range is an unbiased estimator of standard deviation [26], whereas the *BWMV* (Equation (7)) is a robust estimator of the statistical dispersion for heavy tailed distributions [27]:

(7) $$\text{BWMV}=\frac{n\overset{n}{\underset{i=1}{\text{∑}}}a_{i}{\left(x_{i}-m\right)}^{2}{\left(1-U_{i}^{2}\right)}^{4}}{{\left(\sum_{i=1}^{n}a_{i}(1-U_{i}^{2})(1-5U_{i}^{2})\right)}^{2}}$$

(8) $$U_{i}=\frac{\mu_{i}-m}{9\text{MAD}}$$

where *m* is the median, *n* is the number of points, *U* is a parameter that comes from Equation (8), *μ* is the mean and *MAD* is the median absolute deviation, being:

(9) $$\text{MAD}=m(\left|x_{i}-m_{x}\right|)$$

where *m* denotes de median and *m_x_* is the median of the data. Finally, the value of the parameter *a* (Equation (7)) could be 0 or 1 depending on the *U* value. If −1 ≤ *U* ≤ 1, thus *a* is1; in any case *a* is 0.

## 4. Methodology

**Figure 1.**
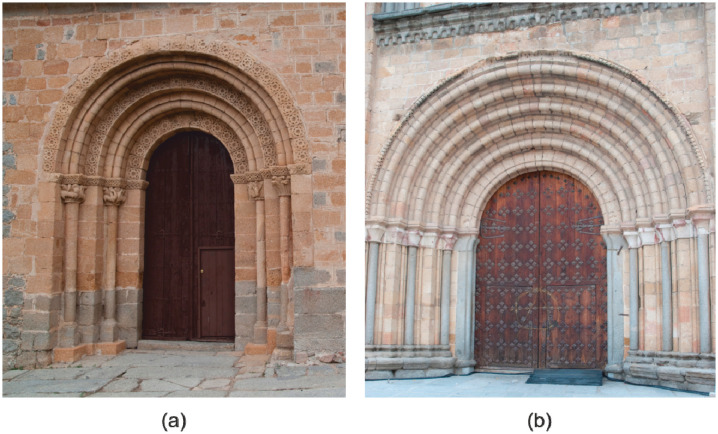
(**a**) Portal of San Segundo; (**b**) Portal of San Pedro.

Two similar sites were chosen to undertake the experiment: the portals of two Romanesque churches—San Pedro and San Segundo—located in the city of Ávila (Spain) (see Figure 1). For these two sites, two data sets with two different instruments, digital camera and terrestrial laser scanner (Table 1) were acquired:

- A double data set by means of convergent photogrammetry, using two different reflex cameras (Canon EOS 500D, Nikon D80).
- A double data set by means of two different types of terrestrial laser scanner, Faro Photon 80 and Trimble GX (Table 1).
- In addition, when processing the photogrammetric data sets, two different tools were used, the commercial software (CS), Agisoft Photoscan, and the PW-Photogrammetry Workbench, the in-house software.

**Figure 2.**
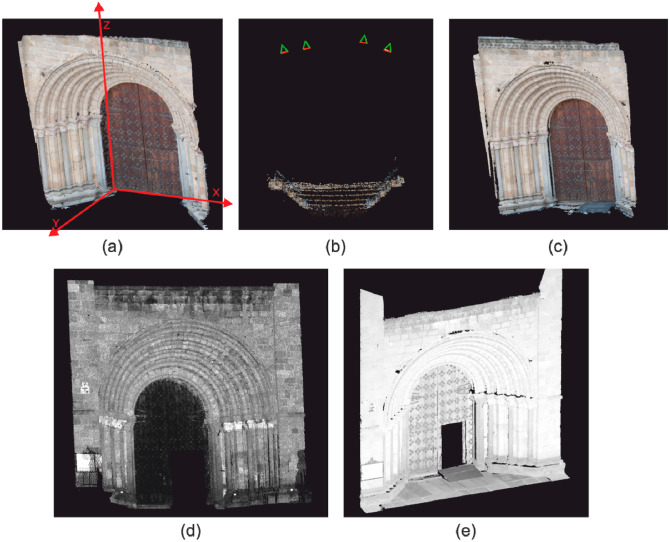
(**a**) Perspective view of the DSM of San Pedro obtained by PW software, showing the local coordinate system; (**b**) Cenital view of the point cloud of San Pedro, showing the photogrammetric shooting geometry; (**c**) Perspective view of the DSM of San Pedro obtained by CS software; (**d**) Perspective view of the DSM of San Pedro obtained by Trimble GX laser scanner; (**e**) Perspective view of the DSM of San Pedro obtained by Faro Photon 80 laser scanner.

This gives us a total of six DSMs for each of the portals: two laser scanner data sets processed once each, and two images data sets processed by two methods each. When comparing photogrammetry *vs.* laser scanner all the photogrammetric DSMs (four per portal) were compared with all the laser scanning DSMs (two per portal) which gives a total of eight comparisons per portal. The camera positions are depicted in Figure 2 along with images of both portals and some examples of the points cloud. To guarantee a common reference frame some control points were accurately observed by means of a total station, Topcon IS Imaging Station.

Both cameras have been chosen for their medium-range performance together with their affordable cost. They provide a 4.7–6 μm pixel size (see Table 1) that guarantees small enough Ground Sample Distances (GSDs): about 3 mm for a focal length of 17–18 mm and a shooting distance of about 10 m. These values, which concern the *a priori* accuracy as well as those that express the *a posteriori* accuracy, are collected on Table 2 and further commented below this table.

The laser scanners are different in their performance: different scanning speed and different vertical field of view (see Table 1). Both sensors are also different in their accuracy: 1.4 mm at 50 m and 2 mm at 25 m. (see Table 1). For the specific case of the two portals data acquisition, the *a priori* accuracy values (as well as the *a posteriori* values) are collected on Table 2 and further commented immediately below.

The total station was chosen for its performance and high accuracy. In any case, this last issue is not very important since its role was to provide a unique coordinate frame (see Figure 2) for all the data sets so that they could be compared to each other. In other words, it is the relative (and not the absolute) accuracy which is relevant here.

The software to process the laser scanners point clouds was Trimble Realworks for a time of flight laser scanner and Faro Scene for the phase-shift sensor, whereas the georeferencing of the points clouds according to the total station coordinates was performed in Helios in-house software (own developed software).

Finally, it should be remarked that both portals exhibit an a priori radiometric good behaviour. The surfaces materials are wood or stone with rich density level patterns so that matching algorithms can perform well. Figure 1 below shows examples of both set of images whereas Figure 2 shows examples of some of the Digital Surface Models obtained.

Table 2 shows values of the *a priori* and *a posteriori* accuracy of the datasets. For the *a priori* photogrammetric accuracy, the Ground Sample Distance (GSD) is assumed for planimetry (*XZ* plane) while GSD*D/B is assumed for the relief direction (*Y* axis), where *D* is the average distance along the *Y* axis between camera stations and object and *B* is the maximum distance between camera stations. For the *a priori* laser scanning accuracy, the Reshetyuk equation [28] is used. For the photogrammetric case, a posterior accuracy, the sigma naught of the bundle adjustment “projected” on the object (by means of the quotient between the GSD and the pixel size) is used. For the *a posteriori* laser scanning accuracy, the root mean square error (RMSE) of geo-referencing of the point cloud with the Ground Control Points (GCP) is used. In order to provide a good assessment of the degree of agreement between DSM from photogrammetry and laser scanning the following procedure was assumed:

(1) To match the points of the laser scanning DSM to the points of the photogrammetric DSM a minimum distance approach was applied.
(2) Once a pair of points was set, the difference of the three coordinate components was evaluated. This threefold strategy is due to the fact that a 2.5D photogrammetric structure configuration must be assumed rather than a 3D one and a different behaviour for the fundamental plane *XZ* and for the relief direction (*Y*) must be expected. *X* is the width dimension of the portal, Z is the height of the portal; *Y* is the depth of the portal.

## 5. Experimental Results

As a previous step and in order to assess the agreement between photogrammetric results and laser scanner results, the non-parametric correlation coefficients were computed, by means of Spearman's correlation coefficient. On Table 3 the results for the Spearman coefficient between data sets for the three coordinates are collected. From them we can see a very high and consistent agreement regarding the *X* and *Z* coordinates (higher for the *Z* case) and a not so high and not so consistent agreement for the *Y* coordinate. These results confirm that photogrammetric and laser scanning derived point clouds are very largely equivalent for the planimetric dimensions (*XZ*), but that along depth dimension (*Y*) the agreement should be addressed much more carefully.

After this, and in order to assess how well the Gaussian and non-Gaussian parameters, discussed on Section 4, describe the differences between pairs of data sets, for both portals, the items that appear on Table 4 (first column) were computed for the three coordinate components (*X,Y,Z*) for each of the comparisons resulting from the laser scanners (Faro Photon 80, Trimble GX) against the photogrammetric approaches (PW, CS) using digital cameras (Canon 500D, Nikon D80). This means that, as described on the previous section, for each pair of data sets (one from scanner laser and the other from photogrammetry) and for each of the three coordinates, a new data set was computed consisting of the difference between matching points from both original data sets. Once obtained this “discrepancy set” the items of the first column were computed by means of statistical tools of Matlab software as well as own implemented statistical software (STAR). Certainly, not all these parameters have the same significance but they are shown here for illustration purposes. Table 4 is an example showing the results obtained for the *Z* component.

The last five rows of Table 4 show the behaviour of gross errors according to the following criteria: *Percentile 0.01* and *Percentile 0.99* collect the observed values that correspond to such percentiles whereas ±2.326*σ, shows the value of observations that lie outside the range of a standard deviation of ±2.326. This threshold is chosen to agree with the percentile 0.01–0.99 criteria, that is, that includes 98% of the sample (or leaves out 2% of the sample). The two last rows show the percentage of observations whose value is smaller than −2.326*σ, or larger than +2.326*σ, respectively (according to strict Gaussian theory it should always be 1%).

**Figure 3.**
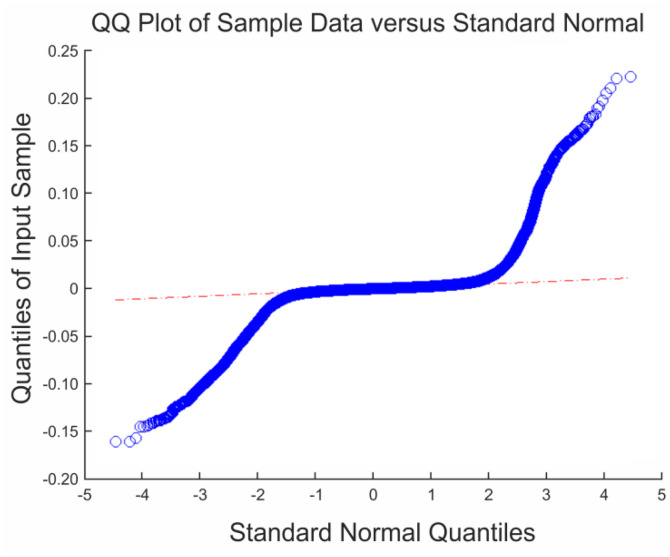
Examples of QQ-plots.

Besides this, the QQ-plots of all of the 48 comparisons were obtained. Figure 3 shows two examples. From the visualization of every one of these plots it could be concluded that there is a large departure from the normal distribution of all the samples due to the presence of gross errors.

In order to assess and discuss the results, the following Tables 5 and 6 show a synthesis of all the comparisons for each of the portals. Each cell gives the average and the [minimum; maximum] interval are presented for the following parameters: Mean (mm), Standard Deviation (mm), Median (mm), Interquartile (Q25–Q75) (mm), Median Absolute Deviation (MAD) (mm), Biweight Midvariance (BWMV) (mm), Kurtosis (adimensional), Skewness (adimensional) and percentage of left (lower) and right (upper) blunders (values larger than ±2.326). From these tables it is possible to highlight the following issues:

The sample mean should show that a certain disagreement is presented between the data sets. For the *X* coordinate in San Segundo, the values are slightly smaller than 1 mm and for the *Z*, the values are even smaller. All the results are very consistent, with small deviations within them and always with the same sign for *X* and *Z*. In the case of San Pedro a systematic effect occurs between data sets that involve Faro or Trimble laser scanner (Table 7). The total displacement between both lasers samples is around 2 mm, affects the width dimension (*X*) and does not appear on the height dimension (*Z*). This question also appears when looking at the median values (see infra) and could be due to an *X* shift when referencing both laser data sets at San Pedro.

Regarding the depth dimension (*Y* coordinate) in the case of San Pedro it can be appreciated that the values are very similar to the planimetric dimensions values, but that the range variation of these values is slightly higher (and, thus, worse) than the planimetric ones. In the case of San Segundo, there appears a difference between 1 and 2 mm between photogrammetry and laser scanner for five of the cases and of 5, 6 and 7 mm for the other three cases which always involve the Trimble laser scanner.

Consequently, although there seems to be a discrepancy between photogrammetry and laser scanner this is too small to declare as significant. In any case, if some systematic trend is appearing it affects the comparative performance between the scanners rather than between the laser scanning and the photogrammetric ones (see Table 7).

Regarding the values of the median, it must be said, first of all, that they always confirm the behaviour that has been stated above concerning the values of mean. The values of the median are sometimes smaller than the values of the mean, sometimes equivalent and sometimes larger but the differences are always very small and always affect the three coordinate components *X*, *Y* and *Z* in a very consistent way.

In addition, under the assumption that the sample median is a more robust estimator than the mean is, the values of this parameter should show a significant disagreement between the data sets but this disagreement is not apparent from the values that have been obtained: the median shows consistently the same small discrepancies that the mean does. Thus, it can be concluded that the existence of blunders or observations that do not conform to the Normal Distribution does not have an influence on the disagreement between data sets. But, as has been said before, the discrepancies are too small to be regarded as significant.

Concerning the values of the standard deviation, it can be seen that for *X* and *Z*, in the case of San Segundo, consistent values around 5 mm are obtained and not as much consistent values around 8 mm are obtained for *Y*. This confirms what is predicted by theory: the precision along the depth direction (*Y*) is worse than for the planimetric coordinates (*X*, *Z*). For these cases (San Segundo), the maximum values (up to 17 mm along *Y* direction) are obtained when using the Nikon camera and the commercial photogrammetric software (CS).

In San Pedro, the standard deviation results are worse than for San Segundo, concerning the *X* and Z coordinates: around 11 mm for the former and around 7 mm for the latter. Also, the consistency is not as high as in the San Segundo case: for both dimensions, the results for the Canon camera are twice as worse than for the Nikon camera. The *Y* results are also worse than the results of San Segundo, about 11 mm, and also show the same differential performance between both cameras.

In any case, the values of the standard deviation are always worse than the a priori values that should be expected from theory. They also show that the *Y* dimension accuracy is worse than the *X-Z* dimensions accuracy at should be expected. This fact is not as clear for San Pedro as it is for San Segundo.

When analysing the percentage of blunders, the first result that must be remarked is that this percentage is 1.6% on average, that is, slightly above what should be expected (1%) and this is not an excessive number of outliers, but much more relevant than this raw and small number is the large variety of results that can be found: from sets that only show 0.05% of blunders to sets where the number of gross errors is 5.4%. Furthermore: there is almost always a significant lack of symmetry between the number of gross errors at the left (lower) and at the right (upper) on the distribution of the frequencies curve. So, what should be highlighted is that it is not a matter of a high number of blunders but of an asymmetric distribution of them.

Confirming what has just been said, all the values that are obtained for the interquantiles as well as for the skewness, show that the data sets do not conform to the symmetry of the Normal curve. It should be remembered that this result is also apparent from the QQ-plots. It should be added that the values of the quantiles (Q25 and Q75) and the median are always consistent with the values of the skewness showing a lack of symmetry to the left or to the right. The consistency is also high for all the comparisons within the same dimension, *X*, *Y* or *Z*.

Finally, when examining the values of the Median Absolute Deviation (MAD) and the Biweight Midvariance (BWMV), very consistent results are achieved for *X* and *Z*, slightly better for *Z* than for *X*. This behaviour (*Z* better than *X*) can be due to the fact that both portals are less complex (in their shapes) along height (*Z*) direction than along width (*X*) direction. It must also be noted that the *X-Z* values are significantly better in the case of San Segundo than in the case of San Pedro. This could be related to the same explanation: San Pedro surfaces are more articulated than San Segundo surfaces. In any case, the values of the Biweight Midvariance for *X* and *Z* lie between 2 and 4 mm and these values certainly meet the a priori expectations. It should be remembered that, on the contrary, the standard deviation values do not meet what the theory predicts.

The *MAD* and *BWMV* results for the depth dimension (*Y* coordinate) are also very consistent within them. They are also very similar between the two portals. These values also meet the a priori expectations and, therefore, also show the relation with the planimetric accuracy values that theory states. In addition, this relation between the accuracy of *X-Z* dimensions and *Y* dimension, expressed through *MAD* and *BWMV*, is much clear than when it is expressed through the standard deviation.

## 6. Conclusions

The DSMs obtained from photogrammetry are largely equivalent to the DSMs obtained from the laser scanner. Some very small inconsistencies have arisen, but these affect the comparative performance of the laser scanners or the comparative performance of the cameras rather than the comparative performance of photogrammetry and laser scanning.

All sets show a large lack of symmetry that leads to the conclusion that the standard Normal parameters are not adequate to assess this type of data. The Normal distribution fails to appropriately describe the data for the cases that have been examined. In particular, this is especially the case when assessing accuracy through the standard deviation, since this parameter fails to provide a good estimation of the results.

Use of non-Normal statistics gives a more appropriate description of the data and yields results that meet what may be expected concerning the assessment of accuracy. The results obtained for the Median Absolute Deviation and for the Biweigth Midvariance agree with the values predicted by the theory.

This can be extended to what usually happens in photogrammetry in a 2.5D case. The planimetric dimensions show better results than the relief (depth) dimension according roughly to the factor *D/B* (distance to the object-camera base quotient). Some results appear to agree with the shape itself of the objects but these values are not apparent enough to consider them a straightforward conclusion.

Regarding future work and in order to extend the validity of these results in the wider field of imaging, this type of experiment could be applied to other cases in which some other shapes, depth variations, images settings or some other network designs (to include real 3D cases) should be analysed. Also, other materials (metal or uniformly painted walls) that should not be as favourable as the ones tested here (wood and stone) must be considered. Of course, more hardware and software should be tested to extend the validity of the conclusions presented here.

## Figures and Tables

**Table 1.** Main characteristics of the instruments that have been used.

**Laser Scanner**		
Model	Trimble GX	Faro Photon 80
Principle	Time of Flight-ToF	Phase Shift-PS
Wavelength	534 nm (Visible - Green)	785 nm (Near Infrared)
Field of view	360 °H × 60 °V	360 °H × 320 °V
Standard deviation	1.4 mm at 50 m	2 mm at 25 m
Measurement Range	2–350 m	0.6–72 m
Spot size (beam diameter)	3 mm a 50 m	8 mm a 50 m
Scanning speed	5,000 points/sec	120,000 points/s
**Reflex Cameras**		
Model	Nikon D80	Canon 500D
Sensor type	CCD (DX format)	APS-C CMOS
Sensor size	23.6 × 15.8 mm	22.3 × 14.9 mm
Resolution	10.2 MP	15.1 MP
Image size	3872 × 2592 pixels	4752 × 3168 pixels
Pixel density	2.7 MP/cm^2^	4.5 MP/cm^2^
File format	12-bit compressed RAW, JPG	14-bit compressed RAW, JPG
**Total Station**		
Model	Topcon IS Imaging Station	
Minimum Reading	1″/5″ | (0.1/0.5 mgon)	
Accuracy	1″, 3″ | (0.3 mgon)	
Tilt Correction	Dual Axis	
Compensating Range	±6′	
Non-Prism (range)	1.5 m 250 m	
Prism (accuracy)	Fine 0.2 mm/1 mm ± (2 mm+2 ppmxD*) m.s.e.	

**Table 2.** *A priori* and *a posteriori* accuracies. For *a priori* photogrammetric accuracies the first term stands for planimetric accuracy whereas the second term stands for relief direction accuracy.

	***A Priori* Accuracy (mm)**	***A Posteriori* Accuracy (mm)**
Canon-CS	2.8/4.8	4.4
Canon-PW	2.8/4.8	3.9
Nikon-CS	3.4/5.8	5.4
Nikon-PW	3.4/5.8	3.7
Faro	3.6	4.2
Trimble	1.7	3.5

**Table 3.** Spearman's correlation between laser scanning and photogrammetric sensors.

**Case**	**Laser**	**Camera**	**Software**	***X***	***Y***	***Z***
San Pedro	Faro	Canon	PW	0.9992	0.9868	0.9997
			CS	0.9998	0.6751	0.9994
		Nikon	PW	0.9998	0.9965	0.9971
			CS	0.9999	0.9948	0.9999
	Trimble	Canon	PW	0.8731	0.6084	0.9328
			CS	0.7981	0.4232	0.9991
		Nikon	PW	0.9998	0.9977	0.9996
			CS	0.9997	0.9849	0.9997
San Segundo	Faro	Canon	PW	0.9702	0.9520	0.9992
			CS	0.9998	0.6219	0.9994
		Nikon	PW	0.9999	0.9993	0.9995
			CS	0.9957	0.8806	0.9987
	Trimble	Canon	PW	0.9999	0.9950	0.9998
			CS	0.9996	0.9178	0.9992
		Nikon	PW	0.9998	0.9857	0.9992
			CS	0.9985	0.8766	0.9985

**Table 4.** Statistical values calculated for the *Z* discrepancies in the case of the portal of San Pedro. *SEM* stands for Standard Error of the Mean. *LCI-UCI*, for Lower-Upper Confidence Interval. *MAD* stands for Median Absolute Deviation. *BWMV*, for Biweight Midvariance. ±2.326*σ, for the range of values of the frequencies Gaussian distribution curve that leaves outside 2% of the sample (see text below for an explanation) *LB-UP*, for Lower-Upper percentage of Blunders (see below for an explanation). All values except Kurtosis and Skewness (adimensional) and percentages are in meters.

	**FARO**				**TRIMBLE**			

	**Canon**		**Nikon**		**Canon**		**Nikon**	
	
	**PW**	**CS**	**PW**	**CS**	**PW**	**CS**	**PW**	**CS**
Sample size (n)	125,379	164,950	138,089	156,154	93,028	128,412	105,628	118,837
Min (m)	−0.1124	−0.1494	−0.0786	−0.0984	−0.1085	−0.1362	−0.1041	−0.1132
Max (m)	0.1742	0.1474	0.0713	0.1294	0.2004	0.1660	0.1104	0.1132
Sample mean (m)	0.0010	0.0011	0.0007	0.0012	0.0007	0.0009	0.0007	0.0009
Standard deviation (m)	0.0084	0.0088	0.0038	0.0045	0.0093	0.0090	0.0056	0.0049
Median (m)	0.0003	0.0006	0.0003	0.0006	0.0001	0.0005	0.0003	0.0005
Quantile 25 (m)	−0.0011	−0.0013	−0.0009	−0.0013	−0.0015	−0.0013	−0.0010	−0.0015
Quantile 75 (m)	0.0019	0.0027	0.0016	0.0027	0.0017	0.0024	0.0016	0.0025
SEM (m)	0.0000	0.0000	0.0000	0.0000	0.0000	0.0000	0.0000	0.0000
LCI of the mean (m)	0.0010	0.0010	0.0007	0.0012	0.0007	0.0008	0.0006	0.0008
UCI of the mean (m)	0.0011	0.0011	0.0007	0.0012	0.0008	0.0009	0.0007	0.0009
LCI of the SD (m)	0.0084	0.0088	0.0038	0.0045	0.0093	0.0089	0.0056	0.0049
UCI of the SD (m)	0.0084	0.0089	0.0038	0.0045	0.0094	0.0090	0.0056	0.0049
MAD (m)	0.0015	0.0020	0.0012	0.0020	0.0016	0.0019	0.0013	0.0020
SQRT(BWMV) (m)	0.0026	0.0035	0.0021	0.0033	0.0028	0.0031	0.0023	0.0031
Percentile 0.1 (m)	−0.0029	−0.0034	−0.0020	−0.0028	−0.0037	−0.0031	−0.0025	−0.0030
Percentile 0.9 (m)	0.0048	0.0075	0.0038	0.0060	0.0045	0.0059	0.0042	0.0049
Kurtosis (adim.)	93.59	29.07	37.11	33.75	104.11	39.79	74.62	60.53
Skewness (adim.)	6.11	−0.02	0.94	1.64	6.89	0.48	2.97	2.72
Percentile 0.01(m)	−0.0159	−0.0272	−0.0073	−0.0073	−0.0168	−0.0274	−0.0120	−0.0092
Percentile 0.99 (m)	0.0288	0.0311	0.0140	0.0162	0.0302	0.0327	0.0175	0.0168
±2.326*σ(m)	±0.0196	±0.0205	±0.0087	±0.0105	±0.0217	±0.0209	±0.0130	±0.0114
LB (−2.326*σ (%)	0.72%	1.66%	0.71%	0.43%	0.65%	1.54%	0.88%	0.65%
UB (+2.326*σ; (%)	1.78%	2.32%	2.78%	3.93%	1.45%	2.11%	1.84%	2.41%

**Table 5.** Synthesis of parametric and non-parametric statistical results for the portal of San Segundo. All magnitudes, except Kurtosis and Skewness (adimensional) and percentages, are in millimetres. *LB-UB* stand for Lower-Upper Blunders (see text for explanation).

	***X***	***Y***	***Z***
Sample mean	−1.0 ∈ [−1.6; −0.4]	2.8 ∈ [−2.0; 7.8]	0.1 ∈ [0.0; 0.5]
Standard deviation	5.4 ∈ [3.6; 7.7]	9.2 ∈ [4.8; 19.3]	4.5 ∈ [2.6; 6.9]
Median	−0.3 ∈ [−0.5; −0.1]	2.4 ∈ [−1.6; 7.7]	0.1 ∈ [0.0; 0.2]
Quantile 25	−1.8 ∈ [−2.6; −1.2]	−0.7 ∈ [−4.3; 3.5]	−1.3 ∈ [−1.8; −1.1]
Quantile 75	1.2 ∈ [0.9; 1.6]	5.9 ∈ [0.6; 12.2]	1.5 ∈ [1.0; 2.0]
*MAD*	1.5 ∈ [1.1; 2.0]	3.3 ∈ [2.2; 4.6]	1.4 ∈ [1.0; 1.9]
√*BWMV*	2.4 ∈ [1.8; 3.2]	5.0 ∈ [3.4; 6.7]	2.3 ∈ [1.8; 3.0]
Kurtosis	61.68 ∈ [18.72; 149.71]	129.48∈ [26.47; 496.93]	125.84∈ [28.22; 311.97]
*LB*	0.28% ∈ [0.05; 0.50]	2.36% ∈ [0.14; 4.08]	1.59% ∈ [0.97; 2.28]
*UB*	2.96% ∈ [1.52; 5.14]	0.73% ∈ [0.11; 1.87]	1.32% ∈ [0.83; 1.59]

**Table 6.** Synthesis of parametric and non-parametric statistical results for the portal of San Pedro. All magnitudes, except Kurtosis and Skewness (adimensional) and percentages, are in millimeters. *LB-UB* stand for Lower-Upper Blunders (see text for explanation).

	***X***	***Y***	***Z***
Sample mean	0.0 ∈ [−2.0; 2.0]	−0.1 ∈ [−1.4; 1.6]	0.9 ∈ [0.7; 1.2]
Standard deviation	11.2 ∈ [5.4; 25.7]	11.9 ∈ [7.0; 24.1]	6.8 ∈ [3.8; 9.3]
Median	0.2 ∈ [−1.0; 2.7]	−0.2 ∈ [−1.5; 2.7]	0.4 ∈ [0.1; 0.6]
Quantile 25	−2.0 ∈ [−3.4; −1.0]	−3.4 ∈ [−5.9; −1.6]	−1.2 ∈ [−1.5; −0.9]
Quantile 75	2.2 ∈ [1.0; 6.8]	2.8 ∈ [1.7; 7.6]	2.1 ∈ [1.6; 2.7]
*MAD*	2.1 ∈ [1.3; 4.4]	3.1 ∈ [1.8; 4.9]	1.7 ∈ [1.2; 2.0]
√*BWMV*	3.7 ∈ [2.4; 8.4]	5.2 ∈ [2.9; 8.1]	2.8 ∈ [2.1; 3.5]
Kurtosis	41.41 ∈ [21.39; 61.42]	101.66 ∈ [18.21; 341.64]	59.07 ∈ [29.07; 104.11]
*LB*	1.56 ∈ [0.21; 2.74]	1.27 ∈ [0.78; 1.91]	2.33 ∈ [1.45; 3.93]
*UB*	1.81 ∈ [0.28; 4.86]	1.49 ∈ [0.30; 2.98]	0.91 ∈ [0.43; 1.66]

**Table 7.** Systematic effect that appears between both laser data sets concerning the width dimension (*X*) in San Pedro. The systematic trend involves the mean and the median. Values in millimetres.

	**FARO**				**TRIMBLE**			

	**Canon**		**Nikon**		**Canon**		**Nikon**	
			
	**PW**	**CS**	**PW**	**CS**	**PW**	**CS**	**PW**	**CS**
Sample mean	−1.3	−1.3	−1.0	−2.0	1.3	2.0	1.5	0.6

Median	−0.1	−0.4	−0.2	−1.0	0.2	2.7	0.4	0.0
